# Massa Valvar Mitral em Paciente com Suspeita de Lúpus Sistêmico: Tumor, Endocardite ou Ambos?

**DOI:** 10.36660/abc.20200206

**Published:** 2020-12-01

**Authors:** Thiago Sant’Anna Coutinho, Bárbara Cristina Rodrigues de Almeida, Guilherme Dalcol Torres de Amorim, Monica Zappa, Clara Weksler, Cristiane da Cruz Lamas

**Affiliations:** 1 Instituto Nacional de Cardiologia Rio de JaneiroRJ Brasil Instituto Nacional de Cardiologia, Rio de Janeiro, RJ - Brasil; 2 Instituto Nacional de Infectologia Evandro Chagas Centro Hospitalar Rio de JaneiroRJ Brasil Instituto Nacional de Infectologia Evandro Chagas - Centro Hospitalar, Rio de Janeiro, RJ – Brasil; 3 Universidade do Grande Rio Rio de JaneiroRJ Brasil Universidade do Grande Rio, Rio de Janeiro, RJ - Brasil

**Keywords:** Valva Mitral/cirurgia, Valva Mitral/patologia, Diagnóstico por Imagem, Ecocardiografia, Ressonância Magnética, Mixoma Infectado, Endocardite, Lupus Eritematoso, Neoplasia Cardíaca

## Abstract

Apresentamos o relato de caso de uma paciente com mixoma valvar mitral infectado e uma revisão da literatura sobre o assunto. Uma mulher de 33 anos apresentou histórico de febre e dispneia com evolução de alguns dias. Na hospitalização, ela apresentava uma síndrome semelhante ao lúpus, com hemoculturas positivas para *Haemophilus spp* . O ecocardiograma revelou uma massa gigante envolvendo ambos os folhetos mitrais associada à regurgitação grave, necessitando de troca valvar mitral biológica. A microscopia revelou mixoma infectado e a paciente recebeu alta assintomática após o término da antibioticoterapia. Ela apresentou bons resultados no seguimento. Este é o sexto caso de mixoma valvar mitral infectado relatado na literatura e o terceiro caso de mixoma cardíaco infectado pelo grupo HACEK. Devido à alta incidência de eventos embólicos, a antibioticoterapia precoce aliada à pronta intervenção cirúrgica são decisivos para a redução da morbimortalidade. O tempo para o diagnóstico foi muito mais breve do que o geralmente relatado em casos de endocardite por HACEK. A troca valvar foi a intervenção mais comum e todos os pacientes em relatos de caso anteriores apresentaram bons resultados no seguimento.

## Introdução

Os mixomas de válvula cardíaca são extremamente raros.^1^ A tríade de sintomas constitucionais, obstrutivos e embólicos torna o diagnóstico diferencial com endocardite desafiador. Excepcionalmente, os próprios mixomas podem estar infectados.

## Métodos

É relatado o caso de uma paciente com mixoma valvar mitral infectado por *Haemophilus spp* . Uma busca nas bases de dados Medline e Lilacs foi realizada desde a primeira publicação sobre o tema até 2019 para fins epidemiológicos.

## Resultados

**Figura 1:**
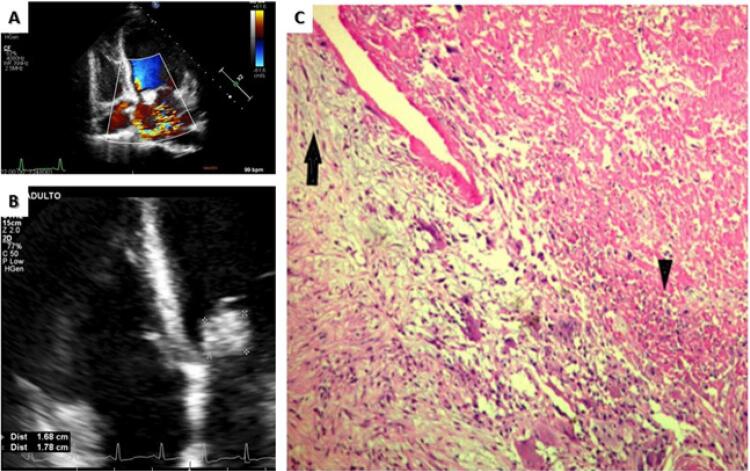
– A) Ecocardiografia com corte apical de quatro câmaras demonstrando insuficiência mitral grave. B) Ecocardiografia com corte apical de quatro câmaras. C) Coloração com hematoxilina-eosina 40x, o mixoma é visto na área azul formada por células estreladas em um estroma mixóide (seta), com infiltração de neutrófilos e necrose (ponta de seta).

Mulher de 33 anos, previamente hígida, apresentou em dezembro de 2017 dispneia progressiva, febre alta, sudorese noturna e perda de peso. Após um mês de evolução, ela foi internada em um hospital geral em franca insuficiência respiratória e choque séptico com infiltrados alveolares difusos, icterícia, hemoptoicos e petéquias nos membros inferiores. Ela foi intubada e precisou de suporte hemodinâmico. Um leve sopro sistólico mitral foi identificado à ausculta do precórdio. Havia leucocitose acentuada com desvio à esquerda, plaquetopenia, disfunção hepática e renal associada à proteinúria subnefrótica e consumo de complemento. O resultado dos anticorpos antinucleares foi de 1/80, apesar dos níveis normais de anti-DNA de fita dupla, anti-SM e anti-PR3. Após a administração de ceftriaxona, ela melhorou clinicamente. Febre amarela, dengue, Chikungunya, leptospirose, HIV e hepatites virais foram descartados. As hemoculturas foram positivas para *Haemophilus spp.* em todas as seis amostras coletadas. O ecocardiograma transtorácico (ETT) demonstrou uma massa ecogênica amorfa com superfície irregular e alguns elementos móveis que envolviam ambos os folhetos da válvula mitral, medindo 20x17 mm no folheto anterior e 19 mm em seu maior diâmetro nos folhetos posteriores, resultando em regurgitação grave por *flail* mitral e perfuração ( Figura 1 ). A ressonância magnética mostrou pequenos abscessos esplênicos, tratados de maneira conservadora. Um aneurisma micótico não-complicado da artéria cerebral média esquerda foi tratado por embolização percutânea. Trinta dias após a hospitalização, ela foi submetida à substituição da válvula mitral com sucesso por uma prótese valvar biológica Sorin^® ^tamanho 29mm e após extensa ressecção do tumor. Foi evidenciada regurgitação aórtica moderada devido a lesão da fibrosa intervalvar mitroaórtica e retração da cúspide não coronariana, tratada de maneira conservadora. O exame patológico confirmou a presença de um mixoma valvar mitral infectado ( Figura 1 ). A paciente completou 28 dias de ceftriaxona e gentamicina, recebendo alta hospitalar assintomática. No seguimento de um ano, não havia evidência de recorrência e constatava-se somente regurgitação aórtica leve. Mixomas infectados apresentam maior risco de eventos embólicos, embora as manifestações clínicas sejam indistinguíveis de tumores não infectados.^2^ O presente caso parece ser o sexto de mixoma valvar mitral infectado relatado na literatura, preenchendo critérios definitivos para o diagnóstico, e o terceiro causado por um microrganismo do grupo HACEK ( Tabela 1 ).^3 - 8^ Dos 64 mixomas valvares mitrais publicados de 2006 a 2012, os sintomas eram cardiovasculares em 36,7%; 9,5 a 21,6% dos mixomas da válvula mitral foram submetidos à troca valvar e o tempo do diagnóstico até a cirurgia variou de algumas horas a 42 dias.^2 , 9^ A mortalidade operatória e geral foi relatada como sendo, respectivamente, 2,6 a 3% e 5,1 a 21%.^2 , 10^ Na presente série, a maioria dos pacientes apresentou insuficiência cardíaca sintomática, foram submetidos à troca valvar mitral e todos apresentaram bons resultados no seguimento.

**Tabela 1:** – Mixomas valvares mitrais infectados descritos nas bases de dados MEDLINE e LILACS

Ref.	Autor	Ano/ País	Sexo/ Idade (anos)	Micro-organismo	Diagnóstico	Apresentação	Localização/Cirurgia	Complicações pós-operatórias	Desfecho
**PR**	**Coutinho **	2020 Brasil	F/33	*Haemophilus spp.*	Eco Critérios definitivos	Sintomas constitucionais Choque séptico e insuficiência respiratória Regurgitação mitral grave Abscesso esplênico Aneurisma micótico	Folheto anterior Tumor 20x17mm Ressecção do tumor e troca valvar mitral biológica	*Flutter* atrial (imediata) Regurgitação aórtica moderada a grave (tardia)	Sobreviveu NYHA I Sem recorrência Seguimento de 1 ano
**(8)**	**Ghazi **	1988 Reino Unido	F/17	*Haemophilus parainfluenzae*	Eco Critérios definitivos	Sintomas constitucionais Vômito, diarreia e dor abdominal Sepse Insuficiência mitral	Folheto posterior Tumor de 10mm Ressecção do tumor e anuloplastia	Sem intercorrências	Sobreviveu NYHA I Sem recorrência Seguimento de 9 meses
**(7)**	**Mrozinski**	1997 Polônia	F/4	*Staphylococcus aureus*	Eco Critérios definitivos	Sintomas constitucionais Insuficiência cardíaca aguda Regurgitação mitral grave	Ambos os folhetos Tumor 30mm Ressecção do tumor e substituição por válvula mitral mecânica	Sem intercorrências	Sobreviveu NYHA I Sem recorrência Tempo de seguimento não está claro
**(6)**	**Toda**	1999 Japão	M/20	*Hemoculturas negativas Bactérias observadas na patologia*	Eco Critérios definitivos	Sintomas constitucionais Síncope Oclusão arterial aguda de membro Regurgitação mitral moderada	Folheto posterior Tumor 20mm Ressecção de tumor e cordas, troca valvar mitral mecânica	Sem intercorrências	Sobreviveu NYHA I Sem recorrência Seguimento de 2 anos
**(5)**	**Liu**	2005 China	F/12	*Neisseria lactamica*	Eco Critérios definitivos	Sintomas constitucionais Insuficiência cardíaca aguda Regurgitação mitral grave	Folheto anterior Tumor de 35x25mm Ressecção do tumor e músculo papilar, troca valvar mitral mecânica e cirurgia de revascularização do miocárdio	Sem intercorrências	Sobreviveu Sem dados sobre a classe funcional Sem recorrência Seguimento de 6 anos
**(4)**	**Guler**	2007 Turquia	F/12	*Staphylococcus aureus*	Eco Critérios definitivos	Início agudo de febre, suor e fadiga Sepse Regurgitação mitral leve	Folheto anterior Tumor 29x18mm Ressecção de tumor	Rotura de aneurisma sacular de aorta abdominal e infarto renal bilateral (tardio)	Sobreviveu Sem dados sobre a classe funcional Sem recorrência Seguimento de 6 meses

*PR: presente relato; F: feminino; M: masculino; Eco: ecocardiograma; NYHA: classe funcional segundo a New York Heart Association.*

## Conclusão

Relatamos um inusitado caso de mixoma de válvula mitral infectado pelo grupo HACEK e complicado por êmbolos sépticos e manifestações imunomediadas. Ao revisar a literatura, descobrimos que a válvula mitral foi mais gravemente danificada pelo tumor infectado comparado aos tumores não-infectados e outros casos de endocardite por HACEK, levando a uma taxa maior de sintomas cardiovasculares e menor tempo para o diagnóstico. Apesar da cirurgia extensa com maior incidência de substituição da válvula em um ambiente de urgência, os pacientes apresentaram bons resultados no seguimento.
