# Supplementary material for: Massa Valvar Mitral em Paciente com Suspeita de Lúpus Sistêmico: Tumor, Endocardite ou Ambos?
Source: Arq Bras Cardiol. 2020 Dec 1;115(6):1201–4. [Article in Portuguese] doi: 10.36660/abc.20200206 (PMC8133720; doi:10.36660/abc.20200206)
Supplement: Translation

# Translation: Mitral Valve Mass in a Patient Suspected of Systemic Lupus: Tumor, Endocarditis or Both?

**Authors:** Thiago Sant’Anna Coutinho, Bárbara Cristina Rodrigues de Almeida, Guilherme Dalcol Torres de Amorim, Monica Zappa, Clara Weksler, Cristiane da Cruz Lamas

**Affiliations:** 1 Brazil Instituto Nacional de Cardiologia, Rio de Janeiro, RJ - Brazil; 2 Brazil Instituto Nacional de Infectologia Evandro Chagas - Centro Hospitalar, Rio de Janeiro, RJ – Brazil; 3 Brazil Universidade do Grande Rio, Rio de Janeiro, RJ - Brazil

**Keywords:** Mitral Valve/surgery, Mitral Valve/pathology, Diagnostic, Imaging, Echocardiography, Magnetic Resonance, Endocarditis, Lupus Erythematosus, Cardiac, Neoplasms

## Abstract

We present a case report of a patient with an infected mitral valve myxoma and a literature review on the subject. A 33-year-old female presented with a history of fever and dyspnea evolving over a few days. On admission, she had a lupus-like syndrome with positive blood cultures for *Haemophilus species* . Echocardiogram revealed a giant mass involving both mitral leaflets causing severe regurgitation, requiring biological mitral valve replacement. Microscopy showed an infected myxoma and the patient was discharged asymptomatic upon completion of antibiotics. She did well on follow-up. This is the sixth case of an infected mitral valve myxoma reported in the literature and the third case of a cardiac myxoma infected by the HACEK group. Exceedingly high incidence of embolic events makes prompt imaging, antibiotic therapy and surgery crucial for better outcomes. Time to diagnosis was much briefer than usually reported in other cases of HACEK endocarditis. Valve replacement was the most common surgical procedure and all patients from previous reports did well on follow-up.

## Introduction

Heart valve myxomas are exceedingly rare.^1^The triad of constitutional, obstructive and embolic symptoms make its differential diagnosis with endocarditis challenging. In the most exceptional scenario, myxomas themselves may be infected.

## Methods

The case of a female patient with an infected mitral valve myxoma by *Haemophilus species* is reported. A search on Medline and Lilacs was conducted from inception to 2019 for epidemiological purposes.

## Results

A previously healthy 33-year-old female presented in December , 2017, with progressive shortness of breath, high-grade fever, night sweats and weight loss. Within a month she was admitted to a general hospital in overt respiratory distress and septic shock with diffuse alveolar infiltrates. Jaundice, bloody sputum and petechiae in the lower limbs ensued. She was intubated and needed hemodynamic support. A soft systolic mitral murmur was heard. There was marked leukocytosis with a left shift, low platelet count, abnormal liver and kidney function tests with near nephrotic proteinuria and complement depletion. Antinuclear antibodies were 1/80 despite normal anti-double stranded-DNA, anti-SM and anti-PR3 levels. After ceftriaxone she improved clinically. Yellow fever, dengue, Chikungunya, leptospirosis, HIV and viral hepatitis were ruled out. Blood cultures were positive for *Haemophilus species* in all six samples collected. Transthoracic echocardiogram demonstrated an amorphous echogenic mass with an irregular surface and a few mobile elements that involved both leaflets of the mitral valve measuring 20x17mm on the anterior leaflet and 19 mm in its greatest diameter on the posterior leaflets, resulting in severe regurgitation by flail and perforation ( Figure 1 ). MRI showed small splenic abscesses, treated conservatively. An uncomplicated mycotic aneurysm of the left middle cerebral artery was managed by percutaneous embolization. Thirty days from admission she underwent successful mitral valve replacement with a biological prosthetic valve Sorin® size 29mm and extensive tumor resection. Moderate aortic regurgitation due to a lesion to the mitroaortic intervalvular fibrosa and retraction of the non-coronary cusp was treated conservatively. Pathology confirmed an infected mitral valve myxoma ( Figure 1 ). The patient completed 28 days of ceftriaxone and gentamicin, being discharged asymptomatic. At one-year follow-up she had no evidence of recurrence and only mild aortic regurgitation. Infected myxomas present a greater risk of embolic events, though clinical features may be indistinguishable from uninfected tumors.^2^The present case appears to be the sixth reported infected mitral valve myxoma by fulfilling previously published definitive criteria and the third caused by a microorganism from the HACEK group ( table 1 ).^3 - 8^Of 64 mitral valve myxomas published from 2006 to 2012, symptoms were cardiovascular in 36,7%; 9.5 to 21.6% of mitral valve myxomas underwent valve replacement and the timing until surgery varied from a few hours to 42 days.^2 , 9^Operative and overall mortality was reported to be respectively 2.6 to 3% and 5.1 to 21%.^2 , 10^In the present series most patients had overt heart failure, underwent mitral valve replacement and all of them did well on follow-up.

**Figure 1:** – A) Apical four-chamber echocardiographic view demonstrating severe mitral regurgitation. B) Apical four-chamber echocardiographic view. C) Hematoxylin eosin staining 40x, myxoma is seen on the blue area formed by stellate cells in a myxoid stroma (arrow), with neutrophil infiltration and necrosis (arrowhead).

**Table 1:** – Infected mitral valve myxomas described in MEDLINE and LILACS

Ref.	Author	Year/ Country	Sex/ Age(y/o)	Organism	Diagnosis	Presentation	Location/Surgery	Postoperative complications	Outcome
PR	Coutinho	2020 Brazil	F/33	Haemophilus spp.	Echo Definitive criteria	Constitutional symptoms Septic shock and respiratory distress Severe mitral regurgitation Splenic abscess Mycotic aneurism	Anterior leaflet 20x17mm tumor Tumor resection and biological mitral valve replacement	Atrial flutter (immediate) Moderate to severe aortic regurgitation (late)	Survived NYHA I No recurrence 1-year follow-up
(8)	Ghazi	1988 UK	F/17	Haemophilus parainfluenzae	Echo Definitive criteria	Constitutional symptoms Vomiting, diarrhea and abdominal pain Sepsis Mitral regurgitation	Posterior leaflet 10mm tumor Tumor resection and annuloplasty	Uneventful	Survived NYHA I No recurrence 9-month follow-up
(7)	Mrozinski	1997 Poland	F/4	Staphylococcus aureus	Echo Definitive criteria	Constitutional symptoms Acute heart failure Severe mitral regurgitation	Both leaflets 30mm tumor Tumor resection and mechanical mitral valve replacement	Uneventful	Survived NYHA I No recurrence Unclear follow-up time
(6)	Toda	1999 Japan	M/20	Negative blood cultures Bacteria seen on pathology	Echo Definitive criteria	Constitutional symptoms Syncope Acute limb arterial occlusion Moderate mitral regurgitation	Posterior leaflet 20mm tumor Tumor and chordae resection, mechanical mitral valve replacement	Uneventful	Survived NYHA I No recurrence 2-year follow-up
(5)	Liu	2005 China	F/12	Neisseria lactamica	Echo Definitive criteria	Constitutional symptoms Acute heart failure Severe mitral regurgitation	Anterior leaflet 35x25mm tumor Tumor and papillary muscle resection, mechanical mitral valve replacement and urgent bypass graft surgery	Uneventful	Survived No data on functional status No recurrence 6-year follow-up
(4)	Guler	2007 Turkey	F/12	Staphylococcus aureus	Echo Definitive criteria	Acute onset of fever, sweating and fatigue Sepsis Mild mitral regurgitation	Anterior leaflet 29x18mm tumor Tumor resection	Ruptured saccular abdominal aortic aneurysm and bilateral renal infarction (late)	Survived No data on functional status No recurrence 6-moth follow-up

*PR: present report; F: female; M: male; N/D: no data; Echo: echocardiogram; NYHA: New York Heart Association functional class.*

## Conclusion

This case of a mitral valve myxoma infected by the HACEK group and further complicated by septic emboli and immune-mediated manifestations is most interesting. On literature review, we found that the mitral valve was more severely damaged by the infected tumor compared to uninfected tumors and to other cases of HACEK endocarditis, leading to a higher rate of cardiovascular symptoms and shorter time to diagnosis. Despite extensive surgery with greater incidence of valve replacement in an urgent setting, patients did well on follow-up.
